# Bioarchaeological insights into the last plague of Imola (1630–1632)

**DOI:** 10.1038/s41598-021-98214-2

**Published:** 2021-11-15

**Authors:** Meriam Guellil, Natascia Rinaldo, Nicoletta Zedda, Oliver Kersten, Xabier Gonzalez Muro, Nils Chr. Stenseth, Emanuela Gualdi-Russo, Barbara Bramanti

**Affiliations:** 1 Centre for Ecological and Evolutionary Synthesis (CEES), Department of Biosciences, University of Oslo, 0316 Oslo, Norway; 2 Institute of Genomics, Estonian Biocentre, University of Tartu, 51010 Tartu, Estonia; 3 Department of Neuroscience and Rehabilitation, Faculty of Medicine, Pharmacy and Prevention, University of Ferrara, 44121 Ferrara, Italy; 4 Department of Archaeology, University of Bologna, Bologna, Italy

**Keywords:** Anthropology, Archaeology, Metagenomics

## Abstract

The plague of 1630–1632 was one of the deadliest plague epidemics to ever hit Northern Italy, and for many of the affected regions, it was also the last. While accounts on plague during the early 1630s in Florence and Milan are frequent, much less is known about the city of Imola. We analyzed the full skeletal assemblage of four mass graves (n = 133 individuals) at the Lazaretto dell’Osservanza, which date back to the outbreak of 1630–1632 in Imola and evaluated our results by integrating new archival sources. The skeletons showed little evidence of physical trauma and were covered by multiple layers of lime, which is characteristic for epidemic mass mortality sites. We screened 15 teeth for *Yersinia pestis* aDNA and were able to confirm the presence of plague in Imola via metagenomic analysis. Additionally, we studied a contemporaneous register, in which a friar recorded patient outcomes at the lazaretto during the last year of the epidemic. Our multidisciplinary approach combining historical, osteological and genomic data provided a unique opportunity to reconstruct an in-depth picture of the last plague of Imola through the city's main lazaretto.

## Introduction

**Figure 1:**
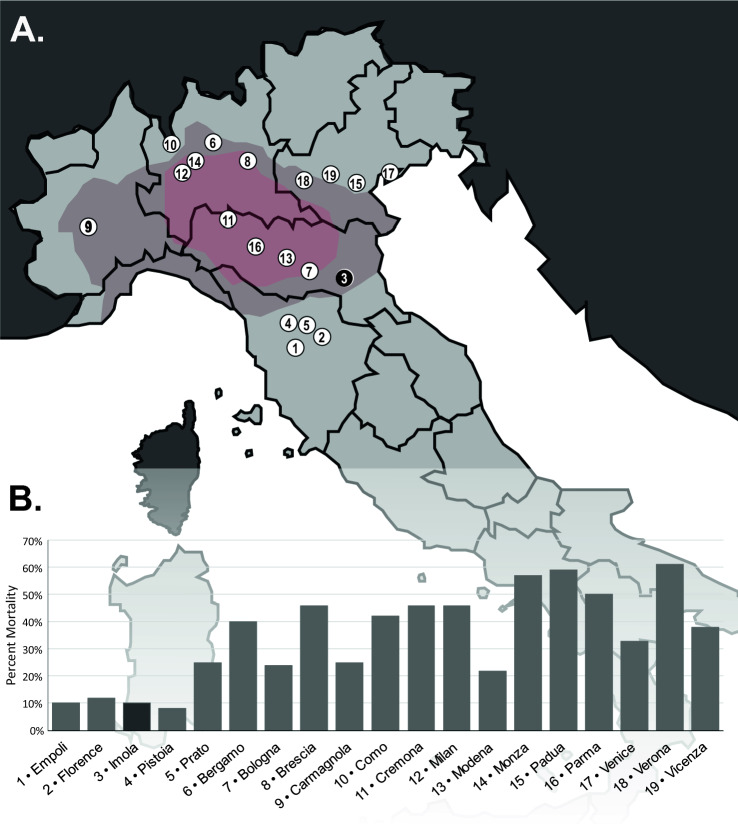
The plague of 1630–1632 in Northern and Central Italy. (**A**) Map of Northern and Central Italy showing the cities recorded on (**B**). Areas known to have been affected by the famine of 1628–1629 are marked in red and areas speculated to have been affected in grey-red. The site of Imola is marked in black. Vector basemap from Wikimedia Commons (Carte ITALIE R1, 2018). (**B**) Mortality rates for cities marked in (**A**) (as reported in Cipolla 2012). The mortality rate for the city of Pistoia was revised according to the data detailed in Cipolla 1981. (The vector basemap was modified from “Carte ITALIE R1” by Wikisoft on Wikimedia Commons 5 licensed under CC BY-SA 3.0 license).

Imola is a city located in the Italian province of Bologna and the region Emilia-Romagna. The city, which was formerly also known as Forum Cornelii during Antiquity, was founded around 82 BCE along the Via Emilia by the river Santerno and was an important urban centre during the Italian Renaissance. Imola was hit by a pestilence during the Italian Plague of 1629–1631, which had reached Northern Italy via soldiers engaged in the Thirty-Year War^1,2^. The plague epidemic started in Milan, where first cases of plague were reported in mid-October 1629. Shortly after, French troops also introduced plague to the Piedmont region^1^. The disease then proceeded to ravage through Northern and Central Italy^3^. In 1630 alone, 34 Italian cities were affected by the disease^3^. In fact, the seventeenth century marks one of two periods of demographic regression for Italy, with the second being after the Black Death during the 1300s^4^. Throughout the Northern Italian plague epidemics of 1629–1631 and the Southern Italian plague epidemics of 1656–1657, a total estimated 20% of the Italian population was lost^4^. Northern Italy was hit particularly heavily, with mortality rates ranging from 20 to 60% (see Fig. 1). Preceding typhus epidemics and famines in 1628 and 1629 are speculated to have weakened the population’s immune defense prior to the arrival of plague^1^.

First reports of an epidemic in Imola were recorded on the 29th of June 1630^2^, when the disease reached the city from Bologna despite the implementation of dedicated quarantine and safety measures. While contemporary historical texts describe buboes in Imola^2^, no molecular evidence for the presence of the plague pathogen *Yersinia pestis* during these epidemics has been available thus far. As plague does not leave identifiable skeletal lesions on the bones of infected individuals, ancient DNA (aDNA) analysis is the only available method to confirm the presence of *Y. pestis* in archaeological samples unequivocally. We apply a multidisciplinary approach to study the plague outbreaks in Imola between 1630 and 1632 by analysing the remains discovered in four mass graves excavated at the Lazaretto dell’Osservanza and by integrating data from an unpublished register detailing the outcomes of patients, which were treated at the lazaretto at the time of the outbreak.

### Archaeological and historical background

The site of “Osservanza” (Imola, Italy) was a clerical complex situated south of the medieval city walls close to today’s Montanara gate. It was founded in July 1466 and had been operated by Observant Friars since 1468^6^. During the epidemic of 1630–1632, the complex was used as a lazaretto for the care of infected individuals from the city and the surrounding countryside, and as a burial ground for the deceased^2^. According to historical records, “more than 100” mass graves were dug at the lazaretto near the old bed of the river Santerno in 1630, each of them accommodating 8–10 cadavers^2^.

Survey excavations were carried out at the site Osservanza under the scientific direction of the Superintendence for Archaeological Heritage of Emilia-Romagna between January and February 2007. The excavated area was divided into two sectors (A and B). Sector A yielded five east–west oriented graves within trenches II and III^7^, which were discovered 0.90 m below the modern walking horizon. All excavated burials were mass graves, each containing 19 to 64 individuals, except for one single grave containing a female skeleton. This individual was omitted from this study as we have no direct evidence of its contemporaneousness to the mass graves.

**Figure 2:**
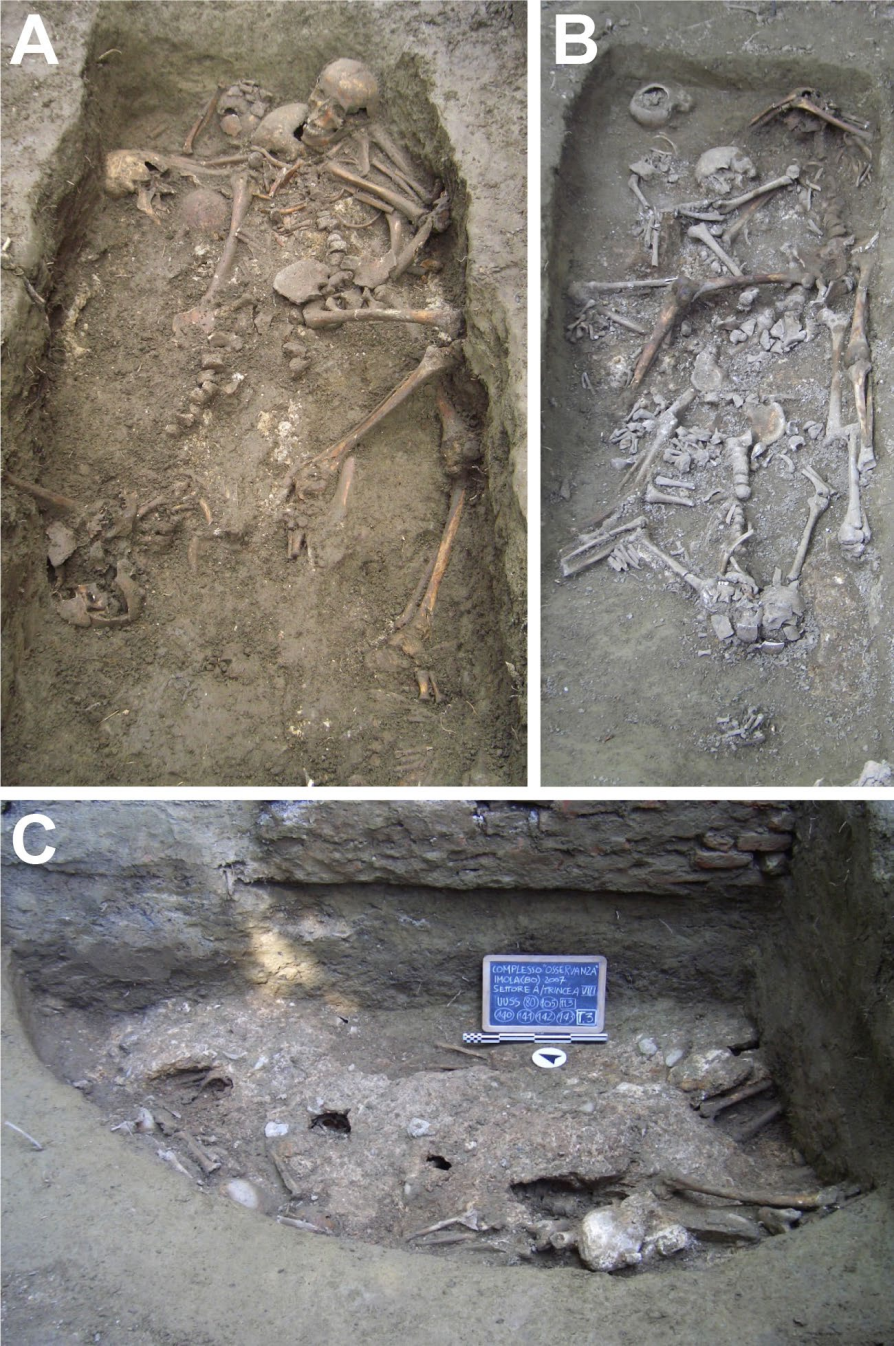
Pictures of grave 6 (**A**), grave 8 (**B**) and grave 3 (**C**) excavated in sector A of the Imola Osservanza complex (BO) in 2007. The use of lime, which was spread in multiple layers in between skeletons, is clearly visible in picture C (These pictures were previously published in Rinaldo et al.^7^, Photos by Xabier Gonzalez Muro).

The excavated mass graves contained a total of 133 skeletons, which are currently stored at the Laboratory of Archaeo-Anthropology and Forensic Anthropology at the Department of Neuroscience and Rehabilitation (University of Ferrara). The skeletons were arranged in a disjointed and disorganized way with east–west and west–east orientations (Fig. 2). The archaeological record suggests that the victims were buried in a single deposition event. Strikingly, multiple layers of lime were found in between the skeletons in each mass grave (Fig. 2C), which is indicative of a major epidemic event^8^. The mass-graves were dated using ceramic and coins found in pit holes, which were probably used to discard and burn objects belonging to infected individuals. The excavation yielded two coins from the filling of the pit holes: a bronze coin from 1622 and a silver coin, carrying the names and insignia of a pope named “Gregorius”, which likely refers to Pope Gregory XV, who held the papacy from 1621 to 1623. The ceramic finds could be assigned to the sixteenth and seventeenth century^7^.

According to historical records^2^, the city of Imola had ca. 16,000–17,000 inhabitants prior to the epidemic, during which 1501 individuals were recorded to have died. This translates to a mortality rate of 9–10% in the city and the surrounding countryside. Unlike Northern Italian cities, whose mortality was much higher (Fig. 1), Central-Northern cities affected by the same epidemic show similar mortality rates. During the first wave of the epidemic, from June to November 1630, Imola recorded 128 deaths^9^. The first outbreak was characterized by an increased mortality amongst women, children and particularly the poor^2^, which can be characteristic for an initial wave of pestilence^10^. A second wave of illness started on the 20th June 1631, lasted until December 1631 and caused 509 recorded casualties^2^. Finally, the last wave of the epidemic hit Imola in March 1632, ended in August 1632 and killed 864 individuals^2^.

## Results

### Historical data

In addition to available historical sources^2,9^ , we have gained access to an unpublished register written by friar Francesco Da Gazzo^11^. The register titled “Registro Delle Persone Entrate Nel Lazzaretto dell’Osservanza per La Pestilenza Del 1632.” is available at the Archivio storico comunale di Imola. Da Gazzo recorded the number of infected individuals entering and dying at the Lazaretto “Osservanza” from the 9th of June 1632 to the end of plague in Imola in August 1632. Although this data only comprises the last 3 months of the final year of the epidemic, it provides great insights into the dynamics of the epidemic in Imola and further supports the low mortality reported in other studies of the plague of 1630^12,13^.

In 1632, 1204 individuals were reported to have fallen ill, and 864 individuals are reported to have died of “plague” in the city of Imola. Of these, 69.4% died in the city and 30.6% in the countryside surrounding Imola^2^. Using Da Gazzo’s records^11^ we know that, during the last months of the epidemic, the Lazaretto “Osservanza” housed 54% of the total number of infected individuals recorded in Imola for the year 1632. Of these, 59% are reported to have died. Gender, inferred by the names recorded in the registry, was distributed unequally with 62.2% of admitted infected individuals and 57.3% of deaths being women. While we have no knowledge of the age of adult individuals represented in the register, we know that children, broadly referred to as “putto” in the register, only represented a very small fraction (4.9%) of patients admitted to the lazaretto.

### Osteology

We analyzed all 133 skeletons stemming from the mass graves excavated at the site “Osservanza”. We identified 48 female and 40 male individuals (Table 1), but were unable to confidently determine the biological sex for the remaining 45 individuals, of which 43 were subadults.

**Table 1:** Distribution of individuals recovered from four mass graves at the Lazaretto dell'Osservanza by age and sex.

	Males	Females	Sex not determined	Total indv. count	% of total sample
Infants (0–3 y.)	0	0	10	10	7.5
Children (3–12 y.)	0	0	26	26	19.5
Adolescents (12–20 y.)	7	13	6	26	19.5
Young adults (20–35 y.)	18	17	0	35	26.3
Middle adults (35–50 y.)	10	10	0	20	15
Old Adults (> 50 y.)	4	3	0	7	5.2
Sub-Adults (Age Undefined)	0	0	1	1	0.7
Adults (Age Undefined)	2	4	2	8	6
Total:	40	48	45	133	

Age of death could not be assigned within the defined categories for eight adult individuals, but it was possible to determine the age of death for all but one subadult individual. All age classes were represented (Table 1), as can be expected for epidemic death assemblages^14^. The least represented age classes were infants (8.1%) and old adults (5.6%).

Our paleopathological analysis recorded signs of multiple infectious diseases. Lesions associated with tuberculosis were identified on five individuals: one subadult (8–10 y), three middle adult females and one male classified as young adult. Skeletal changes were frequently recorded on vertebrae, but lesions were also observed on ribs and sternum. Affected vertebrae and ribs showed signs of lytic lesions and cavitation on their vertebral body and on their proximal ends, respectively. However, these lesions are not specific to tuberculosis and could have been caused by other conditions^15^. One young adult male could be a potential case of syphilis, as it displayed signs of periosteal reaction with prominent periosteal striations and cortical thickening, as well as a saber malformation of the tibia, which is a common observation for the treponemal disease^15,16^. One child showed signs of poliomyelitis; the femurs were asymmetrical, and the bone showed signs of malformation due to the premature fusion of the femoral head. We also found evidence for metabolic diseases. We identified potential cases of rickets or osteomalacia on the skeletons of two young adults, which showcased characteristic malformations on their lower limbs. We also recorded a potential case of malignant neoplasia in a middle adult female. We observed a thickening of the individual’s diploe, as well as increased porosity of the internal cranial vault and lytic lesions on the external cranial vault. Similar lytic lesions were also found on the postcranium, particularly on the left clavicle, on some ribs, on the sternum, the femoral neck, and on the os coxae. We could not identify any perimortem traumas, but observed skeletal evidence for antemortem trauma, i.e. trauma exhibiting signs of remodelling, on three adult males and one old adult female.

### Ancient DNA

We sampled teeth from 15 skeletons (Table S1). Of these, IMO2 and IMO7 amplified aDNA using primers for the *Y. pestis* genes *caf1* and *pla* respectively and IMO3a was screened via shotgun sequencing. Based on the PCR results, IMO2a and IMO7a were selected for further metagenomic exploration via shotgun sequencing. Using Kraken2^17^, we found that most reads assigned to *Yersinia* (Table 2) were classified at the genus level or to the so-called “*Yersinia pseudotuberculosis complex*”, which encompasses all *Y. pestis* and *Yersinia pseudotuberculosis* species. Contrary to Kraken2, Metaphlan2^18^ yielded no *Y. pestis* hits for all shotgun datasets (Table 2 and Fig. S2). This is not surprising considering the low number of observed hits using Kraken2. Kraken2, which implements a k-mer based approach, is less sensitive, but incorporates a much bigger database than Metaphlan2, which is limited to unique clade-specific marker genes. This distinction is especially important when considering the misincorporations found in aDNA.

**Table 2:** Results of the metagenomic analysis of shotgun and target enrichment datasets using Kraken2 (Wood et al.^17^) and Metaphlan2 (Segata et al.^18^).

Sample ID	Type of data	Filtered reads	Kraken2							Metaphlan2
			% Reads identified	Identified reads	Yersinia hits	Yersinia PTB complex hits	*Yersinia pestis* hits	Yersinia Pseudotub. hits	% Yersinia hits	% *Yersinia pestis*
IMO2	Shotgun	47,690,216	5.43	2,589,579	743	454	50	11	0.029	0
	Capture Mapped (-n0.01 -q 30)	11,942	89.06	10,636	8694	7743	957	47	81.745	100
IMO3	Shotgun	53,694,249	4.84	2,598,802	126	12	1	3	0.005	0
IMO7	Shotgun	54,138,809	4.26	2,306,313	496	87	4	46	0.022	0

Kraken2 records the number of hits for each taxon and Metaphlan2 the abundance of identified reads assigned to each taxon. Mapped data denotes reads extracted from a mapping to the CO92 reference genome.

Shotgun reads for all three samples were also mapped against the *Y. pestis* CO92 reference sequence (Table 3 and Fig. S5). While all samples had comparable numbers of reads mapping to the reference sequence, sample IMO2, which also yielded the most hits using Kraken2, had the highest number of reads mapping to CO92 above a mapping quality of 30 and ca. 14% of reads displayed the characteristic C > T aDNA deamination at their 5’ end (Fig. S3). Across all three samples, most reads mapped to unspecific regions of the genome. Additionally, IMO2 showed coverage for all plasmids (Table 3). We could not identify clear signs for the presence of *Y. pestis* or any other pathogen within the shotgun datasets from IMO3 and IMO7.

**Table 3:** Mapping statistics for alignments of shotgun datasets to the *Y. pestis* CO92 reference genome (-n 0.01; MQ > 30).

Sample ID	# Mapped reads	% 1X	Edit distance	C > T (5′ Pos. 1)	pCD		pMT		pPCP	
					# Mapped reads	%1X	# Mapped reads	% 1X	# Mapped reads	% 1X
IMO2	753	0.67	0.88	14.23	25	1.66	28	1.47	9	3.92
IMO3	185	0.08	2.11	–	0	0	0	0	1	0.38
IMO7	113	0.07	1.81	–	4	0.25	1	0.05	1	0.45

In order to estimate the overall aDNA preservation across all three samples, we mapped the shotgun data to the rCRS build of the human mitochondrion as well as the Hg38p.12 build of the human allosomes. We recovered 40–100% of the human mitochondrion at mean depths of coverage between 1.4 and 114.7X (Table 4) across all three sequenced samples. The reads mapping to the human mitochondrion also showcased clear signs of deamination (Fig. S4), which combined with the small insert sizes (mean 48.59–69 bp) observed across all samples, is consistent with aDNA. Finally, a thermal age analysis performed for Imola also indicates that the recovery of good quality DNA was possible at the site (λ = 0.0047).

**Table 4:** Mapping statistics for alignments of shotgun datasets to the human mitochondrial DNA rCRS built and the human allososmes (Hg38p.12).

Sample ID	Human DNA (-n 0.1; MQ > 0)			
	mtDNA rCRS % 5X	mtDNA rCRS mean DoC	Haplogroup	Genetic sex
IMO2	100	114.71	H2a2a1	–
IMO3	1.4	1.4	–	Male
IMO7	99.8	38.9	H2a2a	–

Following the screening of shotgun metagenomic datasets, we enriched libraries of sample IMO2 for the plague pathogen *Y. pestis*, using a custom RNA baits design. Unique reads mapping to the *Y. pestis* CO92 reference genome (MQ > 30) were extracted and taxonomically classified using both Kraken2 and Metaphlan2 (Table 2, Fig. S2). We saw a clear increase in hits for the pathogen with up to 81.7% of identified reads being classified as *Yersinia* or to a lower taxonomic rank using Kraken2 and all identifiable reads being assigned to the species *Y. pestis* by Metaphlan2. While we saw an increase of *Y. pestis* hits when compared to the shotgun dataset, the amount of *Y. pestis* sequences recovered via target enrichment still proved inadequate to reconstruct sufficient parts of the *Y. pestis* genomes at a viable depth of coverage. We observed C > T deamination rates of ca. 9.2% at position 1 for the capture mapping (-n 0.01, MQ > 0) (Table 5). Our mapping had a sequence coverage of 16.8% for plasmid pMT1 at a mean depth of coverage of 0.19X, 34.1% for pCD1 at 1.7X and 68.4% for pPCP1 at a mean depth of coverage of 0.44X (Figs. S6 and S7). While low when considering that multiple enriched libraries were used for this assembly, the coverage across plasmids seems to increase with copy number and we could confirm the presence of the *Y. pestis* specific plasmids pPCP1 and pMT^19^.

**Table 5:** Mapping Statistics for alignments of sequencing data from enriched libraries to the *Y. pestis* CO92 reference genome (-n 0.01; MQ > 0).

**(a)**									
IMO2 capture	# mapped reads without duplicates	# Reads > 30q	Mean MQ	C > T (5′ Pos. 1)	G > A (3′ Pos. 1)	Mean Cov	% 1X	% 2X	% 5X
Full reference	27,210	11,942	31.1	9.20%	9.30%	0.3319	14.87	2.84	0.5

**(b)**									
IMO2 capture	# mapped reads without duplicates	# Reads > 30q	Mean MQ	Mean Cov	% 1X	% 2X	% 5X		
Chromosome	26,171	11,048	18.6	0.33	14.4	2.7	0.5		
pCD	501	426	32.3	0.45	34.1	8.7	0		
pMT	286	244	31.9	0.19	16.8	2.2	0.1		
pPCP	252	224	33.6	1.73	68.4	41.3	7.7		

(a) Statistics for whole reference sequence and (b) statistics for each chromosome and plasmid separately.

## Discussion

The results of this study identify the causative pathogen of the epidemic, which affected Imola between 1630 and 1632, as *Yersinia pestis*. In the light of these results and using a multidisciplinary analysis, we evaluated unpublished historical records^11^ in combination with our osteological analysis to provide insights into the epidemic event represented by the four excavated mass graves at the Lazaretto “Osservanza”.

The comparison of our osteological analysis with the register of Francesco Da Gazzo showed some demographic discrepancies, which can probably be attributed to fluctuation in mortality throughout the epidemic. While the number of identified males and females was comparable in the excavated mass-graves (see Tables 1 and 6), the friar’s records show a clear prevalence of female patients and female deaths at the lazaretto at the end of the epidemic. Similarly, we detected a higher-than-expected prevalence of subadult individuals in our assemblage (27%) (see Table 1), relative to the low numbers of child deaths reported in the register (4.9%) (see Table 6). It should also be pointed out that one of the difficulties when comparing the data of the register with our analysis was the definition of the word “putto”, which designated children in the register. It is unclear how broad the age category falling under “putto” is, based on the social norms of the time. The age distribution observed in our data, with a high percentage of children, adolescents and young adults, is typical for a catastrophic assemblage. However, the mortality rates we observed for infants in the mass graves of Osservanza are lower than expected. While this can often be observed in plague assemblages^14,20–22^, the incredibly small number of individuals below the age of three identified in Imola “Osservanza” could also be explained by a separate burial place or grave pit for infants, for example outside of the city, or the existence of another location for the care of sick children. However, the records from the lazaretto of 1632 also show that only 4.9% of admitted patients were children, which seems to coincide with the low incidence of infants identified during our analysis and demonstrates that children were treated at the lazaretto. In light of the bad bone preservation observed in the mass-graves, it is also possible that additional subadult skeletons, which are more likely to be affected by diagenetic processes, were deposited, but not recovered during the excavation. We also cannot exclude the possibility that the mass-graves contained individuals, which had not been treated at the lazaretto.

**Table 6:** Data recovered from the register of friar Francesco Da Gazzo, recording the arrival and death of plague victims at the Lazaretto dell'Osservanza (June–August 1632).

	Infected	Deceased	% Case-fatality ratio
Male	214	143	66.82
Female	405	220	54.32
Children	32	18	56.25
Total:	651	384	58.99

Overall, our palaeodemographic results are consistent with those recorded for other plague sites^20,23,24^. Apart from a few individuals for which we recorded pathogenic markers, the analysed individuals were generally in good health (Table 7). We found little to no evidence of physical trauma, which is typical for epidemic assemblages. In addition to the effects of comorbidities, further analyses on the “frailty” of the individuals recovered from the mass graves of Imola “Osservanza'' should be considered, as they could provide additional evidence for the overall health of the plague victims in Imola by examining the presence of metabolic markers, such as porotic hyperostosis, cribra orbitalia and enamel hypoplasia^25^.

**Table 7:** Frequency of pathologies identified on individual from the excavaed mass-graves at the Lazaretto dell'Osservanza (ND: not determined).

	Infections	Trauma	Neoplasms	Metabolic diseases	Joint diseases
Males	9	5	2	4	7
Females	8	1	2	3	2
ND Sex	3	1	0	4	0
Adults	18	7	4	7	9
Subadults	2	0	0	4	0

The mass-graves excavated at the site of Imola “Osservanza” were dug during the plague epidemic of 1630–1632 to accommodate the burial of large amounts of plague victims, which was confirmed by our metagenomic analysis, which provides evidence for the presence of the plague pathogen *Y. pestis* in the teeth of deposited individuals. The graves excavated during the survey campaign in 2007 seem far from the described 100 organized graves, planned to house no more than ten individuals each. In fact, there is little order within the excavated burials that each contained between 19 and 65 individuals, which were seemingly deposited without particular care in between layers of lime (Fig. 2C). Considering the archaeological records, which certainly only represent a fraction of the plague pits to be expected at the site of “Osservanza”, we could speculate that graves of trench II and III^7^ were dug in 1632, the final and most lethal year of the epidemic. However, the recorded discrepancy in child deaths between the Da Gazzo register and our skeletal assemblage (Tables 1 and 6) probably place the time of death for the individuals discovered in the mass graves prior to June–August 1632 and thus before Da Gazzo started recording the activity at the Lazaretto dell’Osservanza.

The use of lime to cover the cadavers is noteworthy (Fig. 2). Lime was believed to have disinfectant properties, to limit the scavenging of graves by predators and to reduce the odour of decay^8^. It has been documented in a number of known plague pits (eg. Barcelona^26^ and Marseille^20^) and has been shown to considerably slow down the decomposition of cadavers^27,28^. The use of lime has also been shown to have a desiccating effect on low-depth tissue^27^, such as the sampled anatomical region, the cranium. Interestingly, lime was used in multiple layers rather than in one final layer at the site of Imola “Osservanza”, leaving some cadavers effectively protected from the surrounding soil and its biome (Fig. 2). This could have influenced the diagenesis of the buried bodies and might explain some of the variation in aDNA preservation observed within grave 3. Both skeletons IMO3/n2 and IMO7/l2 were recovered from the very bottom of the grave, and were, therefore, completely exposed to the surrounding soil from beneath. These skeletons yielded little to no evidence of the plague pathogen. IMO2/u2, however, was recovered in between layers of lime and on top of other skeletons and yielded low, but detectable, amounts of *Y. pestis* aDNA.

The overall preservation of the skeletal human remains recovered from the Lazaretto “Osservanza'' was poor, with some individuals missing all, but the densest bones of their skeleton upon discovery. However, our mapping to the human mitochondrion shows that while aDNA was not conserved equally amongst the sequenced samples, the overall aDNA preservation was adequate for the identification of bacterial sequences. The sample IMO2, which carried traces of the plague pathogen, also carried the highest amounts of human DNA with its mitochondrial mapping showing a depth of coverage of more than 15X across 99.9% of the sequence. Yet, IMO7a, a sample from which no clear sign for the presence of *Y. pestis* could be recovered, also yielded a high coverage assembly of the human mitochondrion with a good deamination signature, indicating that the overall aDNA preservation of the sampled tooth was good. Beyond environmental and taphonomic factors, the course of disease and the timing/occurence of bacteraemia could have played a role as well.

Much like today, plague probably manifested in various forms throughout an epidemic during the Second Plague Pandemic. However, the low mortality rates recorded in Imola^2,11^, especially compared to other cities affected by plague at the time (Fig. 1), tentatively allows us to exclude the much more lethal pneumonic plague^29^, as the predominant form of the disease spreading through the city and its rural surroundings during the early 1630s. Hence, the population of seventeenth century Imola was very likely affected by bubonic plague. This is further corroborated by descriptions of buboes found in historical text documenting the epidemic^2^. Notwithstanding, other historical sources^2^ hint at possible cases of primary septicaemic plague, as they feature reports of plague victims without buboes.

The third wave of plague in 1632 is characterised by a shift in seasonality. Instead of running from June to November/December, as it had in previous years, the plague hit Imola in March and ended in August. The city of Imola also recorded its highest yearly mortality in 1632, which was 6.7 and 1.7 times higher than in 1630 and 1631, respectively. As Imola was one of only a handful of cities still affected by plague past 1631^30^, there is little available data for comparison. Florence, which also experienced plague in 1632, recorded cases of the disease for the entire year, starting with sporadic cases in January and a peak in mortality between the end of September and the end of December^31^. We investigated if the increase in mortality in Imola for the year 1632 could be related to an increase in birth rates in the previous years, but found that there had been no significant change to the birth rates recorded for the city over the last 5 years^2^. Trends for the region of Emilia-Romagna from 1530 to 1632 also did not show any significant increase except for a peak in 1624 before the region was hit by famine and typhus in 1628–1629^32^. We investigated recorded changes in temperature and humidity and found that, based on a weather diary recorded in Lisbon (December 1630–January 1633)^33^, the spring of 1632 was the wettest period on record with 57 days of rain, resulting in particularly favourable conditions for arthropod vectors^34^.

In conclusion, although we could not detect high amounts of *Y. pestis* via our aDNA analysis, the site of Imola and the conducted multi-disciplinary analyses, have given us the opportunity to describe a multi-facetted plague epidemic. The results of our analysis also point to the possibility of differential aDNA preservation in a grave containing multiple layers of lime, which could be valuable information for designing a sampling strategy in similar graves. Although further data will be needed to validate this hypothesis. As we only screened 12% of individuals excavated from Imola “Osservanza” for the presence of plague, further metagenomic exploration of individuals recovered from the mass-graves could yield sufficient data for a phylogenetic analysis in the future.

## Material and methods

For this study, a total of 133 individuals were analyzed osteologically. Of these, 15 individuals were chosen for ancient DNA (aDNA) analysis. We extracted two teeth from nine skeletons (IMO1, IMO3–IMO10), one tooth from five subadult skeletons (IMO11–15) and seven teeth from skeleton IMO2 (Table S1). Sampled teeth were either molars or premolars, which were still embedded in alveolar bone and exhibited dental wear equal or below stage 4 as defined in Smith and Kight^35^. Selected teeth also had to be free of dental pathologies and cavities.

We sampled at least two individuals per mass grave, except for grave 3, from which four individuals were selected as it contained the largest amount of skeletons. Individuals were chosen from the top and bottom layers of the graves, ideally separated by at least one layer of lime. The teeth were sampled under clean conditions at the Laboratory of Archaeo-Anthropology and Forensic Anthropology, Department of Neuroscience and Rehabilitation in Ferrara, Italy. They were then sealed and sent off to the aDNA laboratory of the University of Oslo for further analysis. To avoid contamination of the skeletal material, the analysis was performed using protective clothing such as coveralls, masks and gloves at all times.

### Osteology

Sex and age-at-death were assessed by morphometric methods. Estimation of the age at death for immature individuals was estimated primarily based on the degree of dental maturation and dental eruption, diaphyseal length of the long bones and stage of ossifications. For adults, we determined the age-at-death by analysing age-related changes to the pubic symphysis and the auricular surfaces of the os coxae. Whenever these anatomical sites were missing, we based our analysis on dental wear and the ossification of the ectocranial sutures of the cranial vault^36–49^. Sex diagnosis was performed only for adults and late adolescents (≥ 15 years of age) using the morphological traits of the skull and the pelvis and metric traits if these bones were not available^50–52^. The samples were assigned to the following age classes after Buikstra and Ubelaker^36^: fetus (pre-natal), infant (0–3 yrs old), child (3–12 yrs old), adolescent (12–20 yrs old), young adult (20–34 yrs old), middle adult (35–49 yrs old), old adult (50 + yrs old). Pathologies were also recorded to provide further background information. The morphological examination was based on a macroscopic analysis.

### Ancient DNA laboratory work

The aDNA analysis was performed in a dedicated aDNA laboratory at the University of Oslo, Department of Biosciences. The introduction of contamination into the workspace is, amongst other measures, minimized by the use of protective clothing, including Tyvek suits, gloves, masks, helmets/visors and hairnets. Benchtops and other surfaces in the lab are routinely wiped down with soap, bleach and/or DNA ExitusPlus. Additionally, the lab is equipped with filtered ventilation, positive airflow and ceiling UV lights. All reagents and equipment in the aDNA laboratory are dedicated solely to the study of aDNA. No modern samples have ever been processed in the laboratory.

#### Sample preparation

After the introduction into the laboratory, samples were UV irradiated for 30 min on each side in a dedicated UV cupboard. The outer 1–2 mm surface of the teeth was then mechanically removed using a dental Renfert Basic quattro IS sandblaster. After another cycle of UV irradiation, the samples were milled to powder using a Retsch Oscillating Mill.

#### Extraction

We used two protocols to extract DNA from the Imola samples. Samples IMO1-10ab and samples IMO11-15a were extracted using protocol A. Samples IMO2d-g were extracted using protocol B.

Protocol A: 0.120 g of tooth powder was extracted using a modified version of the extraction protocols published by Dabney et al.^53^ and Bennett et al.^54^. 1 ml of lysis buffer (0.25 mg/ml Proteinase K, 0.45 M EDTA) was added to each sample and incubated on a nutator at 38 ℃ overnight. The lysate was then added to a binding buffer (6X QG Buffer, 4X Isopropanol) and passed through a Qiagen MinElute Spin Column and a Zymo-Spin V 15 ml extension reservoir using a Qiagen Qiavac Vacuum Manifold. The column was washed twice with 1 ml of Qiagen PE Buffer. After a dry spin, the sample was eluted in two steps with a total of 50 µl Qiagen EB Buffer.

Protocol B: 0.20–0.60 g of tooth powder was extracted using a modified version of the protocol published by Brotherton et al.^55^. Samples were digested in 4.3 ml of lysis buffer (0.5 M EDTA, 0.25 mg/mL Proteinase K, 0.5% N-Lauroylsarcosine) on a nutator at 38 ℃ overnight. The lysate was added to 2.5 ml of binding buffer (1X Triton, 0.2 M Acetic Acid, 20 mM NaCl, 13.5 ml of Qiagen QG Buffer) and 120 µl of medium-sized silica suspension and was left to incubate on a nutator for 90 min. The samples were then pelleted, and the silica pellet washed three times with 1 ml of 80% Ethanol. Finally, the dried silica pellets were eluted in 200 µl Qiagen EB Buffer.

#### PCR screening

All samples and blanks controls were screened for the presence of *Y. pestis* by PCR using primers previously listed in Namouchi et al.^56^. Milling and extraction blanks were all negative. For the PCR setup we used Amplitaq Gold Polymerase (1.2 × PCR Buffer, 3 mM MgCl2, 0.2 mM dNTPs Qiagen, 0.4 mg/mL BSA 20 mg/mL, 2.5 U/μl AmpliTaq Polymerase, 0.2 µM per primer, 5 μ of template) with the following conditions: 94 °C for 6 min, 45 cycles of 94 °C/40 s, 60 °C/40 s and 72 °C/40 s. Samples and blank controls were also screened using human mitochondrial HV1 primers L16209/H16348^57,58^ with the PCR setup detailed above. All blank controls were negative.

#### Library preparation

Double-stranded single indexed libraries were prepared according to Meyer and Kircher^59^ with some modifications: (1) a Qiagen MinElute PCR purification kit with Qiagen PB buffer (5X) and one wash with Qiagen PE Buffer was used for all purification steps; (2) following the adapter fill-in step and after the initial 37 °C incubation, samples were incubated at 80 °C for 20 min to denature Bst, and were used for the indexing PCR setup without prior purification; (3) 1.25 µM of adapter mix was used in the adapter ligation master mix; (4) Amplitaq Gold Polymerase was used for the indexing PCR setup.

We set up indexing PCR reactions by splitting 40 μl of denatured adapter fill-in into three reactions and then added 20 μl of indexing PCR master mix (1.2 × AmpliTaq Gold Buffer, 3 mM MgCl2, 0.05 U/μl AmpliTaq Polymerase, 0.4 mg/mL BSA, 200 μM Qiagen dNTPs, 200 μM primer IS4/indexing primer) to each reaction.

Indexing PCR conditions were as follows: 95° for 6 min, 12 cycles at 95 °C/40 s, 60 °C/40 s and 72 °C/40 s and a final elongation step at 72 °C for 10 min. Different indices were used for each tooth. Libraries were purified using Stratec MSB Spin PCRapace columns and Agencourt Ampure XP. Purified libraries were subsequently quantified using an Agilent High Sensitivity Bioanalyzer Chip and a Qubit dsDNA HS Assay.

#### Target enrichment

We enriched libraries from individual IMO2 for the plague pathogen *Y. pestis* with the MYBaits kit from MYcroarray using RNA probes at 3–5 × tiling density and the baits design detailed in Guellil et al.^60^. Before target enrichment, libraries were concentrated to 7 μl using a SpeedVac. All samples were enriched according to the manufacturer’s instructions (MYBaits kit 3.01) with some modifications. During our capture setup, we used half-volume aliquots of MYBaits baits and Block 1–3 for all capture reactions, except IC7 and IC14 for which full aliquots were used.

DNA and baits were hybridized at 55 °C for 40 h for IC7 and IC11. At 60 °C for 30 h for IC14 and IC15 and finally at 65 °C for 27 h for IC18 and IC19.

Subsequently, captured DNA was amplified in duplicate 50 μl PCR reactions using Herculase II Fusion Polymerase, 5 × Herculase II Reaction Buffer, dNTPs (250 μM), primers IS5/IS6 (0.3 μM each) and 15 μl of template under the following conditions: 98 °C for 2 min, 14 cycles of 98 °C for 20 s, 60 °C for 30 s and 72 °C for 30 s, and 72 °C for 5 min. Amplified samples were purified with AmpureXP beads and quantified on a Bioanalyzer 2100 expert chip and with a Qubit ds High Sensitivity Assay.

#### Sequencing

All libraries were sequenced on an Illumina Hiseq 2500 (125 bp PE) system at the Norwegian Sequencing Centre. A total of 176,030,885 (PE) new DNA sequences were generated for this study via shotgun sequencing of WGS libraries and a total of 279,854,787 (PE) were generated via sequencing of enriched libraries.

### Genomic and metagenomics analysis of sequencing data

#### Data preparation and filtering

Raw datasets were demultiplexed at the Norwegian Sequencing Centre, University of Oslo. Using cutadapt, the datasets were trimmed of their adapters and indices. All reads below 30 bp were filtered out, the quality cut-off for read termini was set to 20. Dataset quality was assessed using FastQC^61^.

#### Metagenomic analysis

To remove redundant and uninformative sequences we filtered our shotgun datasets using prinseq-lite^62^. We removed all exact duplicates, as well as all sequences with a dust score above 5. The paired-end reads were then merged using FLASH (-z -M 125)^63^ and analysed using Kraken2^17^ using a custom database containing all dusted bacterial, viral, archaeal and protozoan reference sequences, as well as the UniVec_Core database. The datasets were also analysed using Metaphlan2^64^. Reads mapping to the reference sequence CO92 were extracted and converted to fastq format. We then ran Kraken and Metaphlan2 with the same settings as described above. Prior to the extraction of the reads, duplicates had been removed using Picard’s MarkDuplicates module^65^. Data used for genomic alignments were not filtered using prinseq.

#### Genomics analysis

For both shotgun and captured libraries, trimmed and quality filtered (-q20, > 30 bp) raw reads were merged using FLASH^63^. We then mapped our merged reads to the CO92 assembly of *Y. pestis* (https://www.ncbi.nlm.nih.gov/assembly/GCF_000009065.1) using bwa aln (-n 0.01 -l 1000) and bwa samse^66^. The aligned datasets were then sorted using samtools^67,68^ and duplicates were removed using Picard’s MarkDuplicates module. Statistics were compiled using GATK’s DepthOfCoverage module^69,70^ and Qualimap2^71^. We realigned our reads around Indels using GATK’s RealignerTargetCreator and IndelRealigner modules^69,70^ and computed damage plots using mapDamage2^72^. Captured IMO2 libraries were merged and deduplicated again using Picard’s MarkDuplicates module. Coverage plots were computed using a custom python script.

Additional alignments to the rCRS built of the human mitochondrion (https://www.ncbi.nlm.nih.gov/nuccore/NC_012920) and the allosomal assemblies of the Hg38p.12 built of the human genome (https://www.ncbi.nlm.nih.gov/nuccore/NC_000023.11, https://www.ncbi.nlm.nih.gov/nuccore/NC_000024.10) were performed as described for the *Y. pestis* CO92 reference sequence (-n 0.1 -l 1000). Genetic sex was determined, wherever possible, using a python script as described in Skoglund et al.^73^. Haplotypes were called using Haplogrep2.0^74^ and inspected using PhyloTree^75^.

### Thermal age analysis

We performed thermal age analysis after Smith et al.^76^ on the portal http://thermal-age.eu/. The parameters were set as follows: age of the samples was based on the year 1632, we chose the available DNA depurination (Bone) model, excavation year was set to 2007, year of analysis was set to 2018 and storage conditions were set to 22 ℃ with an annual variation over a range of 15 ℃.

## Supplementary Information

Supplementary Figures.

Supplementary Table 1.

## Data Availability

Sequencing data will be made available upon publication at the European Nucleotide Archive (ENA) under the accession number PRJEB27822.
