# Supplementary material for: Bioarchaeological insights into the last plague of Imola (1630–1632)
Source: Sci Rep. 2021 Nov 15;11:22253. doi: 10.1038/s41598-021-98214-2 (PMC8593082; doi:10.1038/s41598-021-98214-2)
Supplement: Supplementary file 1 — Supplementary Figures. [file 41598_2021_98214_MOESM1_ESM.pdf]

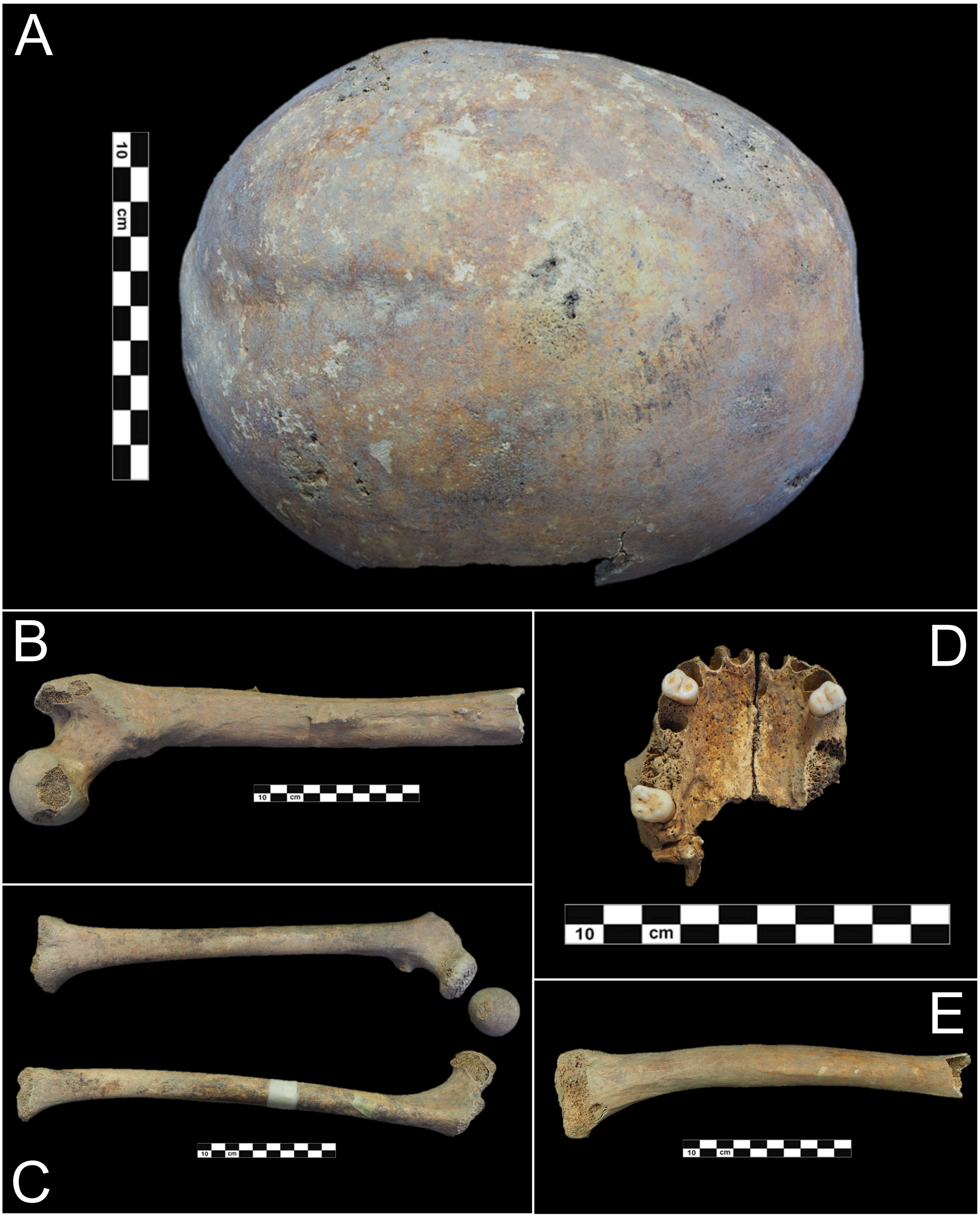

Figure S1: (A) Individual l3 grave 3, lytic lesions on the cranial vault due to metastatic carcinoma. (B) Individual q1 grave 3, traumatic myositis ossificans on the femoral diaphysis. (C) Individual r1 grave 3, premature fusion of the epiphyses and curvature of the left femur due to poliomyelitis. (D) Individual k1 grave 3, osteomyelitis of the maxillary bone. (E) Individual y1 grave 8, curved tibial shaft due to rickets. Photos by Natascia Rinaldo & Nicoletta Zedda.

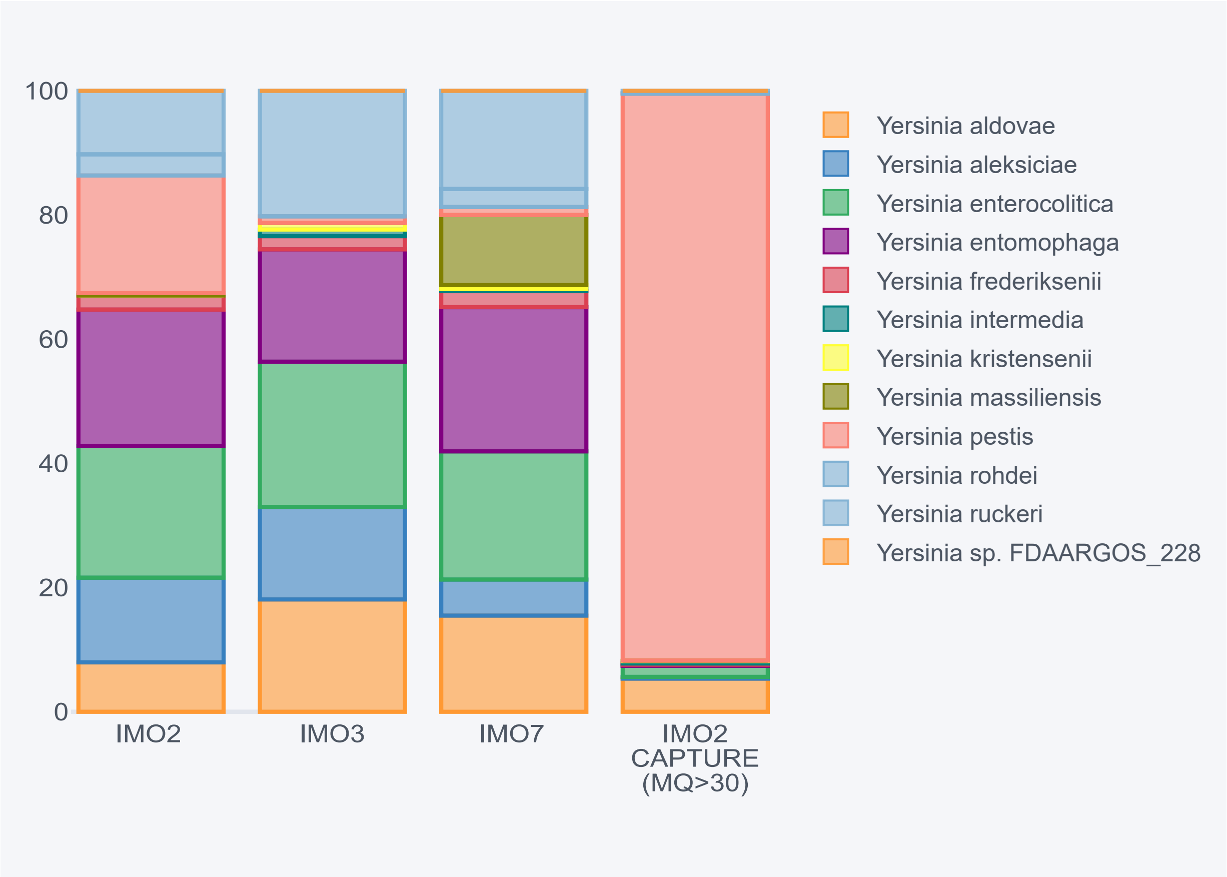

Figure S2: Bar plots showing the percentages of species specific hits within the genus *Yersinia* for each dataset. Data was generated using Kraken2 (Wood et al. 2019) and plots using plotly (Plotly Technologies Inc 2015) in python.

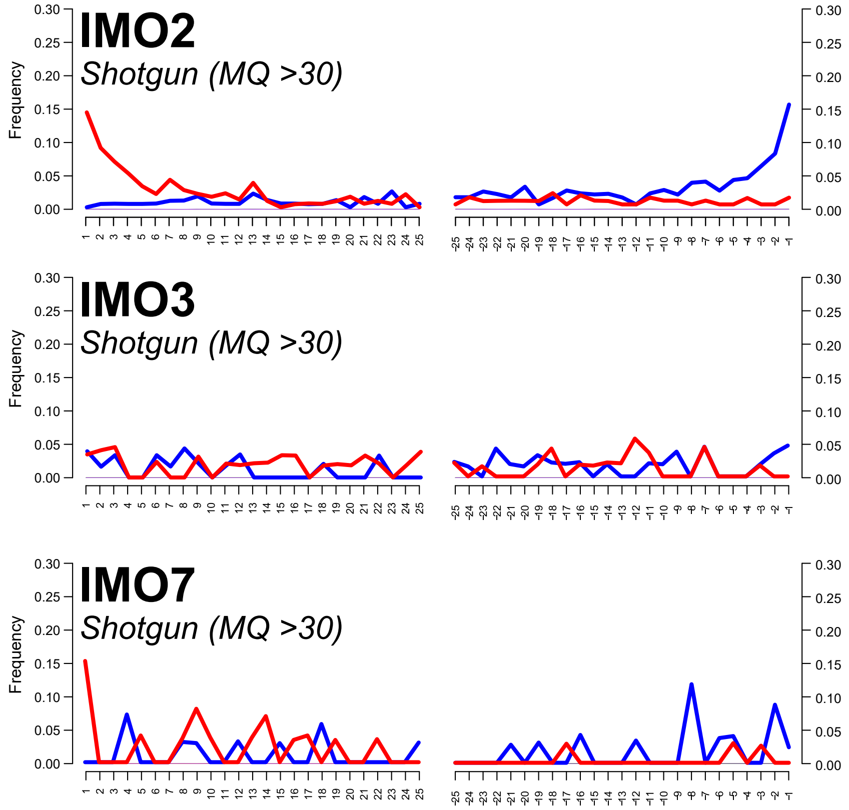

Figure S3: Ancient DNA damage plots for the mapping of IMO2, IMO3 and IMO7 (Shotgun Data) to *Y. pestis CO92* (-n 0.01, MQ>30). The plots show position and frequency of typical aDNA nucleotide substitutions from C to T and G to A, which are reported in red and blue, respectively, and were calculated and visualised using MapDamage2.0 (Jónsson et al. 2013).

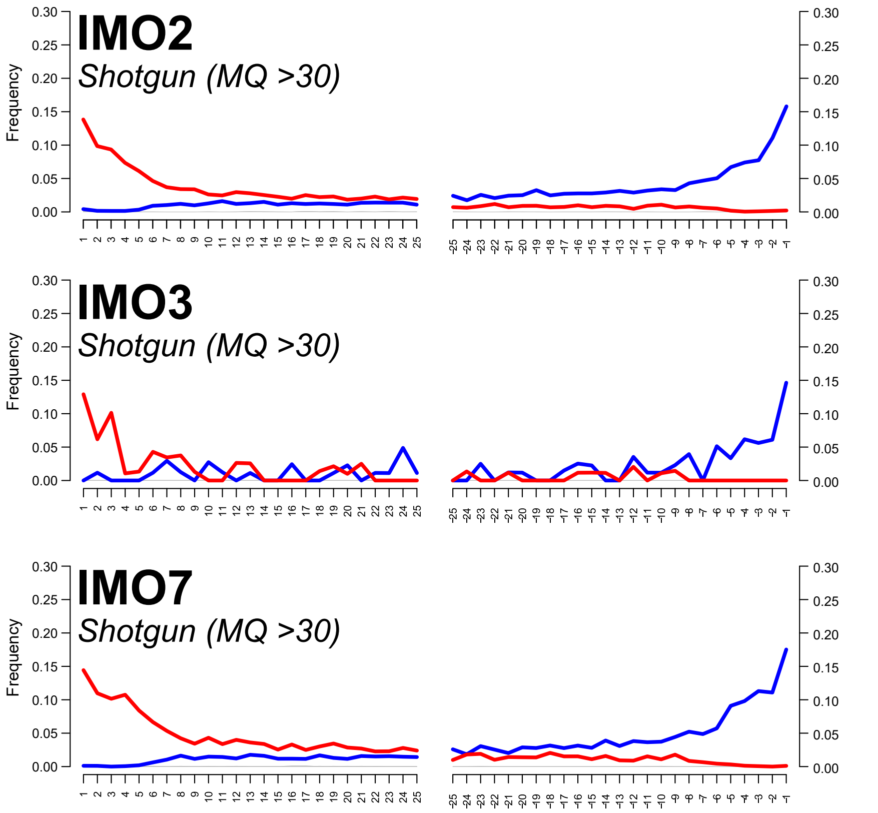

Figure S4: Ancient DNA damage plots for the mapping of IMO2, IMO3 and IMO7 (Shotgun Data) to the rCRS built of the human mitochondrion (-n 0.1, MQ>30). The plots show position and frequency of typical aDNA nucleotide substitutions from C to T and G to A, which are reported in red and blue, respectively, and were calculated and visualised using MapDamage2.0 (Jónsson et al. 2013).

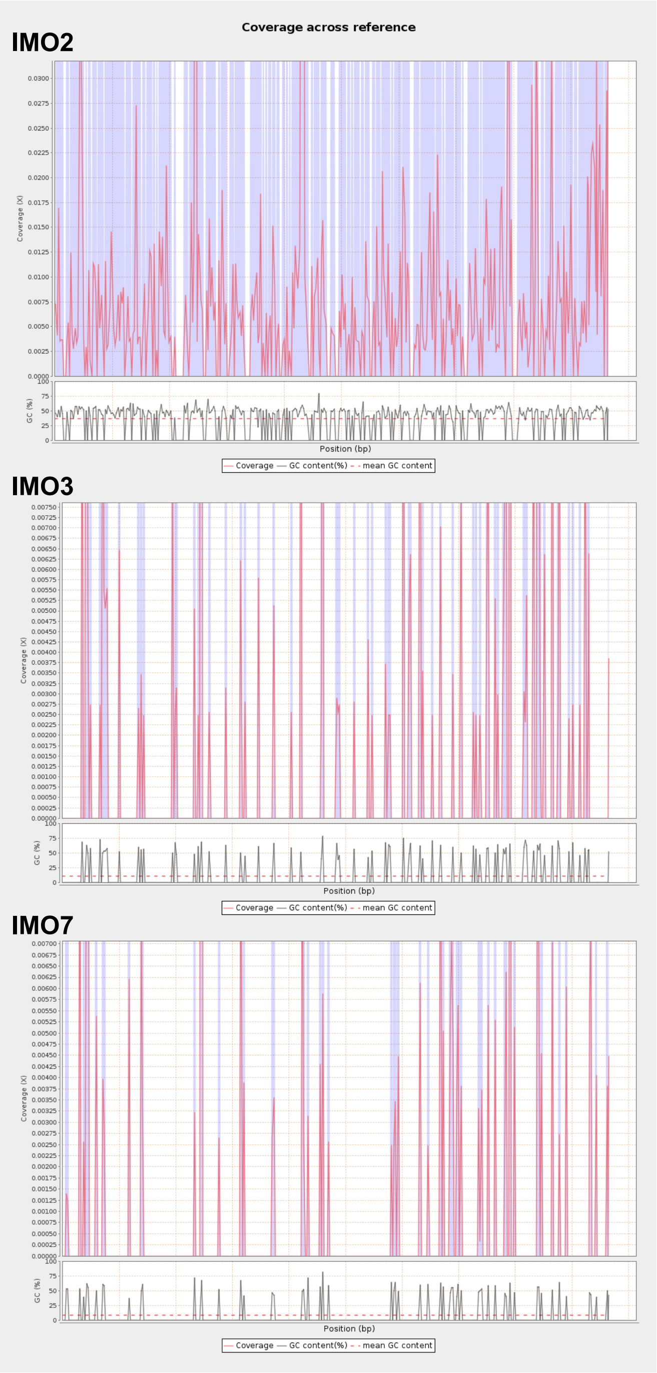

Figure S5: Coverage graphs for the shotgun mappings to the *Y. pestis* CO92 reference genome (-n 0.01; MQ>30). Computed using QualiMap2 (Okonechnikov et al. 2016).

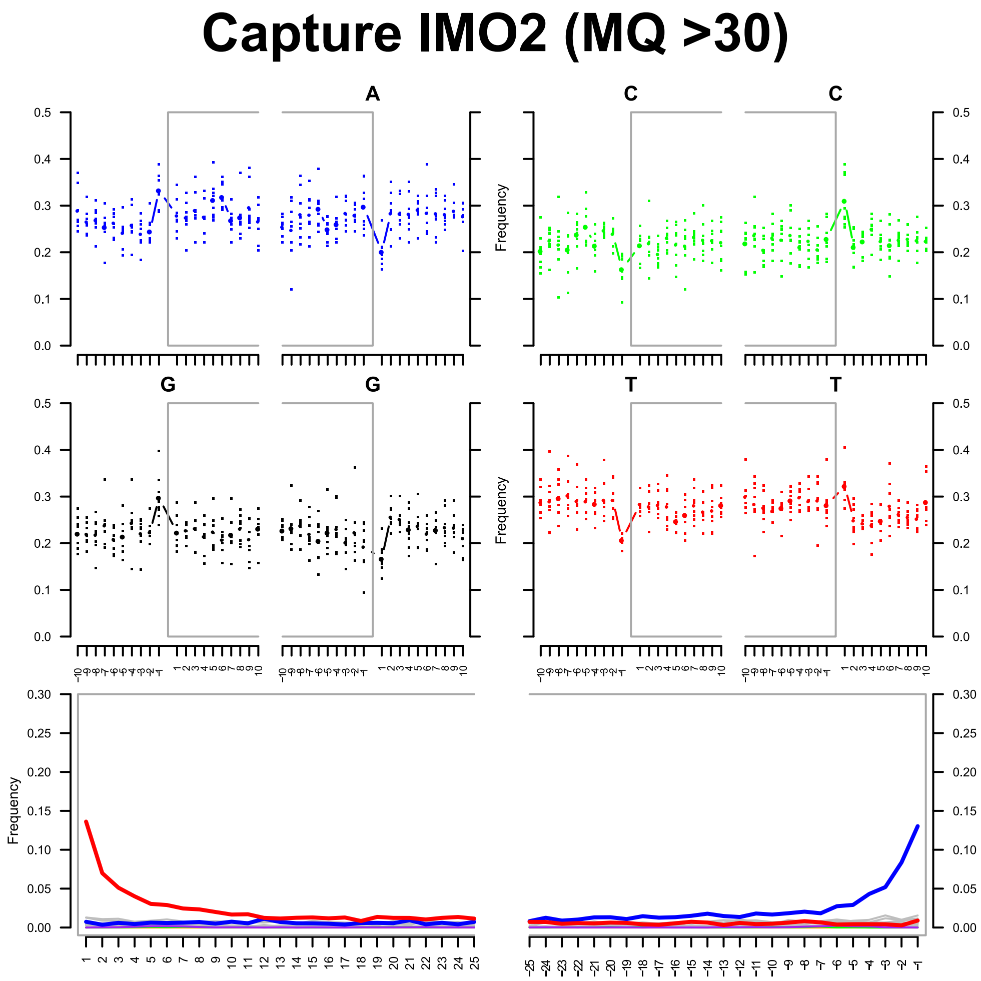

Figure S6: Ancient DNA damage plots for the mapping of IMO2 (Capture Data, -n 0.01, MQ>30) to *Y. pestis CO92*. The four upper mini-plots show the base frequency outside and within the mapped reads (the open grey box corresponds to the read). The bottom plots show position and frequency of typical aDNA nucleotide substitutions from C to T and G to A, which are reported in red and blue, respectively, and were calculated and visualised using MapDamage2.0 (Jónsson et al. 2013).

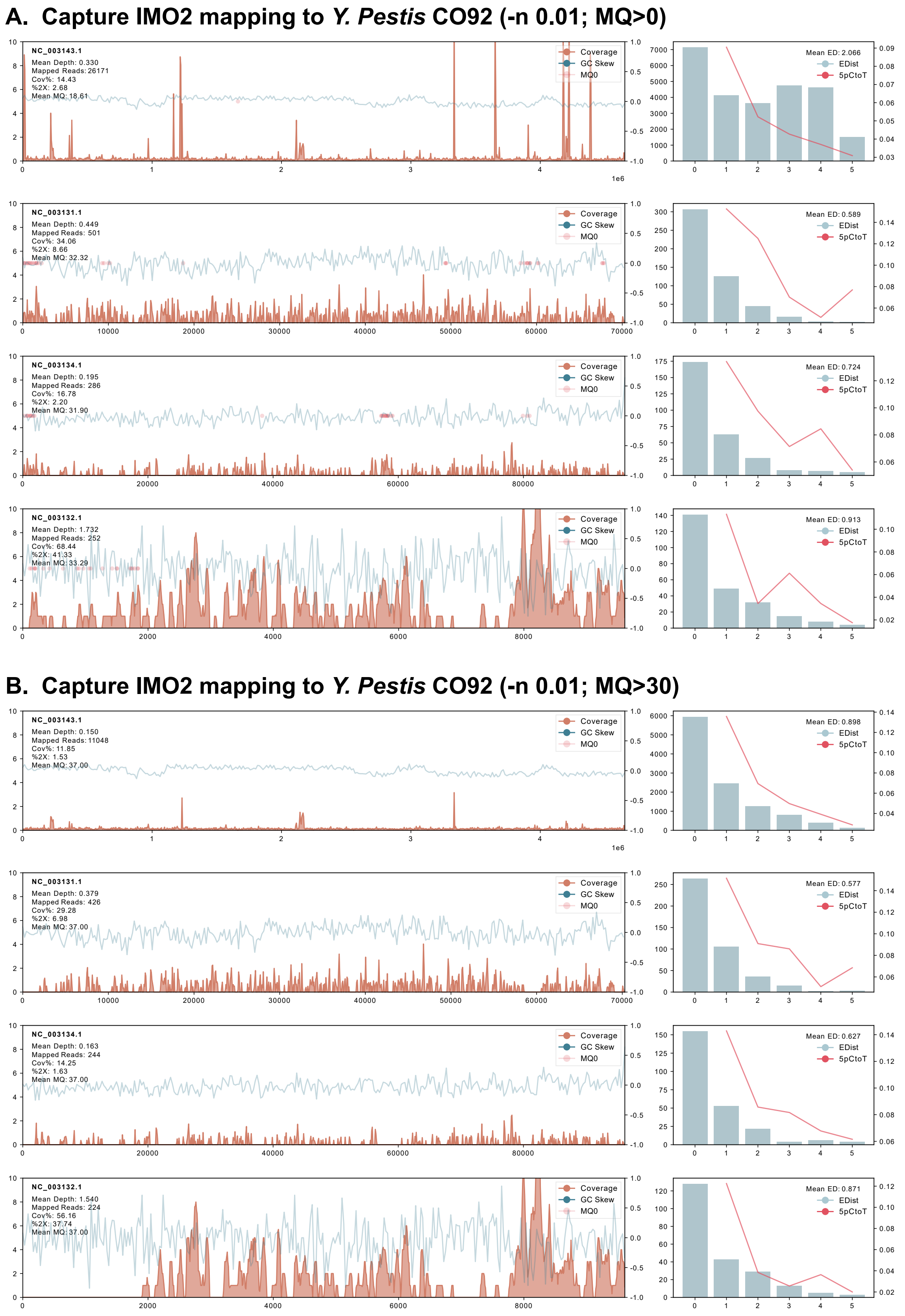

Figure S7: Coverage graphs for the mapped capture data for IMO2 to the *Y. pestis* CO92 reference genome. A) with no mapping quality filter applied and B) with a minimum mapping quality of 30.
